# Tear Film MicroRNAs as Potential Biomarkers: A Review

**DOI:** 10.3390/ijms24043694

**Published:** 2023-02-12

**Authors:** Jeremy Altman, Garrett Jones, Saleh Ahmed, Shruti Sharma, Ashok Sharma

**Affiliations:** 1 Center for Biotechnology and Genomic Medicine, Medical College of Georgia, Augusta University, Augusta, GA 30912, USA; 2 Department of Ophthalmology, Medical College of Georgia, Augusta University, Augusta, GA 30912, USA; 3 Department of Population Health Sciences, Medical College of Georgia, Augusta University, Augusta, GA 30912, USA

**Keywords:** tear film, microRNA, biomarker, ocular diseases, Alzheimer’s and breast cancer

## Abstract

MicroRNAs are non-coding RNAs that serve as regulatory molecules in a variety of pathways such as inflammation, metabolism, homeostasis, cell machinery, and development. With the progression of sequencing methods and modern bioinformatics tools, novel roles of microRNAs in regulatory mechanisms and pathophysiological states continue to expand. Advances in detection methods have further enabled larger adoption of studies utilizing minimal sample volumes, allowing the analysis of microRNAs in low-volume biofluids, such as the aqueous humor and tear fluid. The reported abundance of extracellular microRNAs in these biofluids has prompted studies to explore their biomarker potential. This review compiles the current literature reporting microRNAs in human tear fluid and their association with ocular diseases including dry eye disease, Sjögren’s syndrome, keratitis, vernal keratoconjunctivitis, glaucoma, diabetic macular edema, and diabetic retinopathy, as well as non-ocular diseases, including Alzheimer’s and breast cancer. We also summarize the known roles of these microRNAs and shed light on the future progression of this field.

## 1. Background

MicroRNAs (miRNAs) are a family of short (18–22 nucleotides), non-coding RNAs that serve as significant regulators of overall gene expression [1]. miRNAs released into the extracellular space are capable of providing dynamic insight into cell-to-cell communication, as well as physiological and pathological processes [2,3]. These extracellular miRNAs are detected in biofluids, and their presence in these fluids can be used for diagnostic purposes, giving them significant biomarker potential [4,5,6,7]. miRNAs in circulation have also shown great stability and resistance to degradation over long-term storage and multiple freeze-thaw cycles, which makes them ideal choices for clinical use [8,9]. Furthermore, the nature of miRNAs, with their simpler complexity and straightforward detection/amplification methods, make them a cost-effective choice for the development of established biomarkers compared to their proteomic counterparts [10,11].

Easily accessible biological fluids, such as tears, present exciting potential for future biomarker research. This biofluid is abundant in various molecules and has been shown to contain significantly more unique miRNAs than aqueous humor samples [12]. Advances in miRNA quantitation, paired with the simple, non-invasive collection methods of tears [13], entice mass participant collection [14] and physician engagement [15], which is essential for biomarker validation. Moreover, the importance of studying the relationship between tear composition and ocular health has become more apparent in recent years, and a multitude of studies have reported variations in tear miRNA content across different pathologies [12,16,17,18,19,20,21,22]. The cohesive relationship between miRNAs, the tear film, and health makes tear miRNAs pertinent options as noninvasive and low-cost diagnostic markers for numerous pathologies [13,23,24,25]. Several studies have identified tear miRNAs as potential biomarkers for ocular diseases including diabetic retinopathy [18,26,27], Sjögren’s syndrome [19], glaucoma [20,28], dry eye disease [21,29,30], diabetic macular edema [12], herpes epithelial keratitis [22], and vernal keratoconjunctivitis [31]. Furthermore, tear miRNA alterations have been associated with some non-ocular diseases, such as Alzheimer’s [16] and metastatic breast cancer [32].

**Figure 1:**
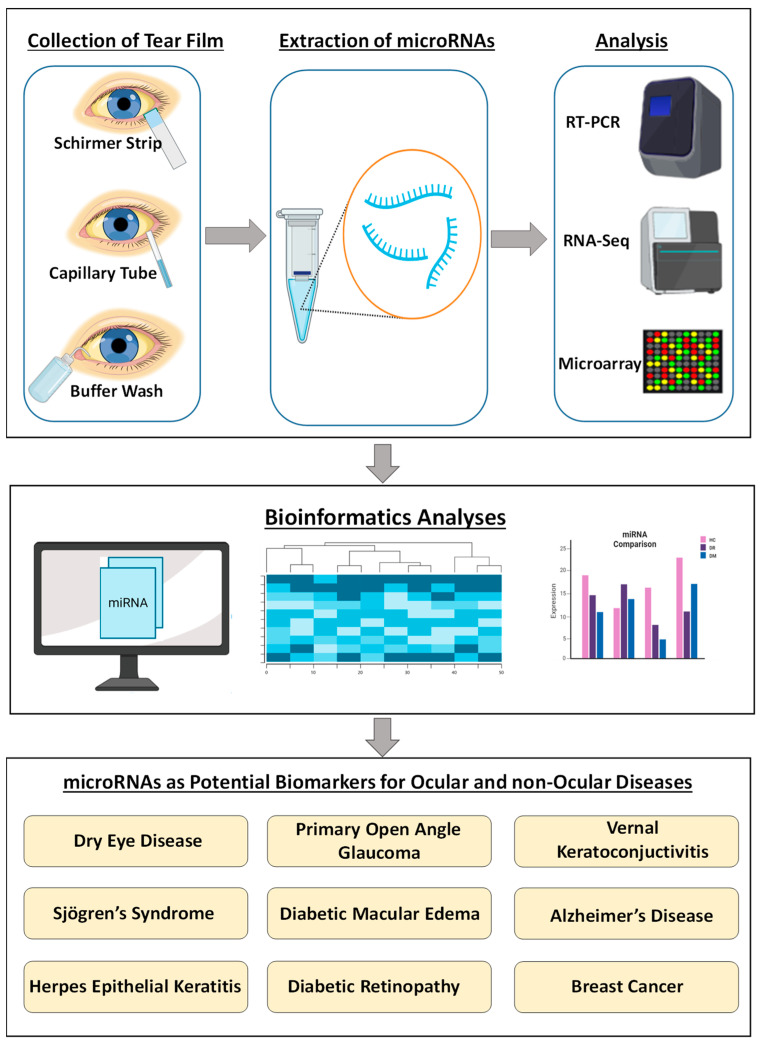
Overall pipeline for the discovery of tear film miRNAs as biomarkers.

This review summarizes the current understanding of miRNAs detected in human tears and their association with both ocular and non-ocular diseases. To date, ~300 unique miRNAs have been reported in tear fluid, of which several are novel and lack in-depth knowledge of their regulatory roles [10,12,16,33]. A comprehensive list of these publications, along with the tear collection and miRNA detection methods used, are presented in Table 1. The general workflow for tear miRNA analysis used in these studies is shown in Figure 1. It is noteworthy that a limited number of studies have examined miRNAs in tears, and therefore more studies with larger sample sets are needed for biomarker validation. 

## 2. Dry Eye Disease

Dry Eye Disease (DED) is a multifactorial disease affecting both the tear film and ocular surface that results in tear film instability and may lead to ocular surface damage [34,35]. The diagnosis of DED could be time-consuming and delayed due to the variability in disease severity, required detailed examinations, and evaluation of history records [36]. Because the tear film is directly altered in this disease, insight into how DED affects its composition can yield novel targets for both diagnosis and treatment.

Searching through the literature, only two studies on profiling tear miRNAs in DED patients have been reported. In one study, nine tear miRNAs (miR-127-5p, miR-1273h-3p, miR-1288-5p, miR-130b-5p, miR-139-3p, miR-1910-5p, miR-203b-5p, miR-22-5p, and miR-4632-3p) were linked to inflammation and were upregulated in the tears of DED patients [29]. The other study reported that four miRNAs (450b-5p, miR-1283, miR-5700, and miR-3671) were significantly upregulated in tears from dry eye patients compared to healthy controls, while 28 tear miRNAs (miR-4673, miR-890, miR-576-5p, miR-337-3p, miR-607, miR-1827, miR-485-5p, miR-6884-5p, miR-335-3p, miR-647, miR-4433b-5p, miR-570-3p, miR-1276, miR-2054, miR-802, miR-539-5p, miR-198, miR-3646, miR-4427, miR-4705, miR-606, miR-20b-3p, miR-4475, miR-4478, miR-4797-5p, miR-142-5p, miR-153-5p, and miR-5583-5p) were significantly downregulated [21]. It is worth noting that there is no overlap in the significantly altered tear miRNAs in DED reported from these two studies. This discrepancy may be attributed to the different pipelines utilized (microarray hybridization vs. RNA-Seq), different collection methodologies (saline wash vs. capillary tubes), and, most importantly, the differences in sample sizes (n = 5 per group: Pucker et al. [29] vs. n = 138 per group: Wang et al. [21]). All of these parameters can contribute to the discrepancies across the literature and examining the impact of each may offer more insight into how to improve reproducibility in future studies exploring this topic. Also, due to high interpersonal variability in tear miRNA levels, studies with a larger sample size are necessary to generate reproducible findings.

Most studies related to these miRNAs (i.e., miR-1288-5p, miR-130b-5p, miR-139-3p, miR-22-5p, miR-203b-5p, and miR-450b-5p) are limited to their involvement in cancer [37,38,39,40,41,42]. One study detected elevated expression of miR-1910-5p in human retinal pigment epithelium cells (APRE-19) in the presence of hydrogen peroxide-induced oxidative stress [43]. Environmental factors, such as exposure to ultraviolet radiation and pollutants, are known to increase oxidative stress and ocular surface inflammation in DED [44]. miR-127, which was elevated in tears from DED patients, has also been shown to promote pro-inflammatory M1 macrophage development in pulmonary tissue of murine models simulating lung inflammation [45]. Higher expression of miR-203 in monkey tears versus sera highlights a potential involvement of miR-203 in ocular homeostasis [46]. Decreased viability of human corneal epithelial (HCE-T) cells has been associated with elevated miR-203 expression compared to basal levels and may be a potential source for its detection in tears [46]. In addition, increased expression of miR-203 in human synovial fibroblasts has been shown to boost the secretion of MMP-1 and IL-6, which are commonly involved in chronic inflammation [47,48,49]. In another study, miR-450b-5p was found to negatively regulate Pax6 in the corneal lineage specification [50]. Pax6 is required for normal eye development [51,52]. However, the precise molecular mechanisms of these miRNAs in DED are still unknown and further studies are required to uncover their roles in an ocular environment.

## 3. Sjögren’s Syndrome

Sjögren’s syndrome (SS) is a chronic autoimmune disorder characterized by lymphocytic infiltration of exocrine glands, especially the salivary and lacrimal glands [53]. Currently, diagnosis typically relies on a combination of patient-reported symptoms of dry mouth/eyes, measurements of ocular and oral dryness, as well as antinuclear antibody blood tests. Many of these signs and symptoms overlap with those of other inflammatory diseases, making diagnosis difficult; often, this delays treatment. The discovery of reliable tear biomarkers of SS can lead to a faster diagnosis and help prevent mismanagement.

In a recent study, four miRNAs were significantly upregulated (miR-16-5p, miR-34a-5p, miR-142-3p, and miR-223-3p) and ten were significantly downregulated (miR-30b-5p, miR-30c-5p, miR-30d-5p, miR-92a-3p, miR-134-5p, miR-137, miR-302d-5p, miR-365b-3p, miR-374c-5p, miR-487b-3p) in patients with SS compared to healthy controls [19]. However, in this study, the expressions of 43 pre-selected miRNAs were measured using a custom PCR array and thus may not encapsulate all the tear miRNAs significantly altered in SS. 

Many of the reported roles of these miRNAs involve the regulation of inflammatory pathways. miR-16 and miR-223 have been reported to be elevated in minor salivary gland tissue and peripheral blood mononuclear cells of SS patients [54]. miR-16 has also been linked to decreased salivary flow rates [55]. Furthermore, the miR-16, miR-142, and miR-223 families are crucial during hematopoietic lineage differentiation and are known to participate in the development and differentiation of immune cells [19,56]. Upregulation of miR-223-3p leads to increased detection of pro-inflammatory cytokines TNF-α and IL-1β in the corneas of mouse models of fungal keratitis [57]. Cortes-Troncoso et al. found that miR-142-3p, which was expressed in the salivary glands of SS patients but not healthy controls, is transported from activated T-cells into glandular cells where it is capable of restricting cAMP production and altering intracellular Ca^2+^ signaling [58]. Both of these processes are critical in fluid and enzyme secretion from exocrine glands [58]. Therefore, the attenuation of elevated miR-223, miR-16, and miR-142 levels may alleviate the heightened immune response in SS patients and relieve symptoms. However, further studies are required to determine their regulatory involvement.

mir-34a has been associated with regulatory networks in T cell activation [59]. The rapid induction of miR-34a expression in T-cell receptors decreased the killing capacity of primary CD4^+^ and CD8^+^ T cells and ultimately shut down the NF-κB signaling process through a proposed feedback loop [60]. Increased activity of CD4^+^ and CD8^+^ T cells are common in peripheral circulation and target tissues of SS patients [61,62], and dysfunction of this miRNA pathway may contribute to the damaged glandular epithelial cells found with severe cytotoxic CD8^+^ T cell infiltration of the exocrine glands and account for the increased miR-34a expression detected in the tears of SS patients. 

Also, miR-92 downregulation is associated with the overexpression of innate defense genes in human corneal epithelial cells in response to excessive inflammation [63]. Furthermore, miR-92a expression in minor salivary gland tissue samples from SS patients and healthy volunteers was inversely correlated with SS severity [64]. However, murine models with elevated miR-17-92 cluster expression in their lymphocytes developed lymphoproliferative disease and autoimmunity with enhanced proliferation of both T and B cells [65]. 

The miR-30 family, which is a known negative regulator of B-cell activating factor (BAFF) [66], was downregulated in the tears of SS patients. BAFF is involved in B-cell maturation, proliferation, and survival, and murine studies have demonstrated that excessive BAFF expression leads to autoimmune-like manifestations [67]. Also, upregulation of BAFF has been observed in B lymphocytes infiltrating the salivary glands of patients with primary SS [66].

## 4. Herpes Epithelial Keratitis

Herpes epithelial keratitis (HEK) is caused by a herpes simplex virus (HSV) infection of the corneal epithelium, which eventually progresses to form dendritic lesions and geographic ulcers [68]. This can result in a loss of corneal sensation, photophobia, and visual impairment due to corneal scarring [69]. 

A recent study analyzed the tear miRNA profile of patients with HEK [22] and found that a total of 23 miRNAs were significantly increased in the tears of patients with HEK compared to controls: miR-15b-5p, miR-16-5p, miR-20b-5p, miR-21-5p, miR-23b-3p, miR-25-3p, miR-29a-3p, miR-30a-3p, miR-30d-5p, miR-92a-3p, miR-124-3p, miR-127-3p, miR-132-3p, miR-142-3p, miR-145-5p, miR-146a-5p, miR-146b-5p, miR-155-5p, miR-182-5p, miR-183-5p, miR-221-3p, miR-223-3p, and miR-338-5p. Furthermore, the expression of miR-29a-3p was found to be significantly increased in the dendritic ulcer group compared to the geographic ulcer groups of the HEK patients [22]. This was only a preliminary study with a small sample size that measured the expression of 43 pre-selected miRNAs in tears from eight patients with HEK and seven age-matched controls. Therefore, the miRNA list presented above may not include all the tear miRNAs changing in relation to HEK.

Increased expression of tear miR-223-3p in HEK patients has also been reported in the corneal stromal cells of fungal keratitis murine models [57], associating it with keratitis pathophysiology. Studies have shown upregulation of miR-155 and miR-132 in association with HSV keratitis murine models, specifically in angiogenesis of the cornea [70,71]. Increased vascular endothelial growth factor (VEGF) and subsequent ocular neovascularization in ocular tissues have been associated with HSV infection [72,73]. Interestingly, VEGF has been shown to upregulate the expression of miR-132 in mice corneas after HSV-1 infection, and in vivo silencing of miR-132 in HSV-infected mice correlated with a reduction in corneal neovascularization [74]. The inactivation of miR-183/92/182 using knockout mice significantly decreased corneal inflammation and the severity of microbial keratitis [75]. Another study reported the involvement of miR-183/92/182 expression on pathways such as IL-17 and IL-10 modulation, TLR4 signaling, and activity of corneal macrophages in miR-183/92/182 knockout mice infected with *P. aeruginosa* that supports the notion of miR-183/92/182 in innate immunity and may contribute to the inflammatory damage experienced in HEK cases [76,77]. 

Significant upregulation of miR-142-3p has been reported in the tears of SS patients as well as in post-transplant corneas of fungal keratitis patients [78]. This elevated expression in corneal tissue may offer insight into a possible source for this miRNA in tears. miR-142-3p has been shown to inhibit the pro-inflammatory TLR-signaling pathway [79] and may be elevated in response to the corneal inflammation caused by HEK. Also, miR-16 has been shown to be involved in Bcl-2 suppression, and the upregulation in tears of HEK patients may be in response to viral-induced apoptosis [80]. The reported increase in expression of miR-29a-3p in dendritic ulcer groups compared to geographical ulcer groups may signal decreased viral replication, as the miR-29a expression has been shown to interfere with viral Nef protein expression and HIV-1 expression in human HEK293T cells [81]. Dysfunction of this pathway may lead to increased viral turnover and would support the notion of continued viral replication leading to the progression of dendritic ulceration into geographical ulceration [82]. Monitoring of this miRNA may provide insight into the development of HEK and exemplifies the importance of proper patient classification in describing disease-staged miRNA fluctuations.

## 5. Vernal Keratoconjunctivitis

Vernal Keratoconjunctivitis (VKC) is a chronic, inflammatory condition of the conjunctiva with a pathogenesis that remains unclear [83]. While the disease is self-limited, its implications can cause corneal scarring and severe visual impairment [84]. A total of 51 tear miRNAs were differentially expressed in VKC patients. The expressions of miR-1229-5p, miR-6821-5p, miR-6800-5p, miR-4466, miR-3665, miR-4530, miR-7110-5p, miR-1207-5p, miR-6875-5p, miR-762, miR-4741, miR-6740-5p, and miR-4298 were significantly upregulated and the expressions of miR-7975, miR-7977, and miR-1260a were significantly downregulated in the VKC patients compared to healthy controls [31]. However, the small sample size (n = 4 per group) may limit the reproducibility of these findings. 

miR-4530 has been associated with the suppression of cell proliferation and the regulation of inflammatory processes [85,86]. The detected upregulation of this miRNA in VKC patient tears may be in response to pro-inflammatory species and stressors present during the disease. Upregulated miR-762 has been shown to negatively regulate host defense genes in human corneal epithelial cells exposed to external antigens [63]. This may contribute to a dysfunctional corneal epithelial layer that allows increased allergen uptake, which can lead to the increased activation of inflammatory responses [87,88]. This study also found upregulated expression of corneal epithelial miR-1207 in response to bacterial antigens, suggesting miR-1207’s involvement in host defense mechanisms. miR-4466 was found to be overexpressed in human corneal stromal cell exosomes of patients with keratoconus compared to healthy controls [89]. This upregulation was also detected in the tears from VKC patients and may indicate miR-4466’s potential involvement in corneal surface integrity.

## 6. Primary Open-Angle Glaucoma

Primary open-angle glaucoma (POAG) is associated with diminished aqueous outflow and cupping of the optic nerve head, which can lead to neuro-retinal degeneration and eventual blindness [90]. When comparing tear miRNA levels in POAG subjects with controls, the expression of miR-26b-5p, miR-27a-3p, miR-152-3p, miR-30e-5p, miR-125b-2-5p, and miR-224-5p was increased in POAG patients, while the expression of miR-151a-3p and miR-1307-3p was decreased [20]. Another study detected decreased levels of miR-146b, miR-126, and miR-16 in the tears of POAG patients compared to healthy controls [28]. It is important to note the variability in miRNAs reported between these studies. Tamkovich et al. [28] pre-selected miR-146b, miR-16, and miR-126 and performed targeted RT-PCR analysis, while Raga-Cervera et al. [20] used RNA-Seq, which would explain the greater number of unique miRNAs reported. Eight miRNAs were significantly altered using RNA-Seq, but the findings of these studies do not overlap. Therefore, other factors such as control group parameters and subject variability may have contributed to this difference.

Many of these miRNAs have been previously associated with glaucoma pathogenesis. miR-125b expression was reported significantly lower in aqueous humor samples of POAG patients [91]. miR-27a, which is believed to have neuroprotective capabilities via the inhibition of proapoptotic Bcl-2 protein synthesis during neuronal cell death [92], was found to be significantly upregulated in glaucomatous rodent retinas [93]. 

Ren et al. have proposed that miR-27a plays a regulatory role in oxidative stress-induced apoptosis of retinal pigment epithelium and retinal degeneration [94]. The miR-27a-induced inhibition of FOXO1-related autophagic functions modulates reactive oxygen species (ROS) accumulation in the retinal pigment epithelium and eventually leads to retinal breakdown [94]. ROS accumulation is a key component in the pathogenesis of POAG [95]. miR-125b, a microglia-enriched miRNA shown to cause constitutive NF-κB activation via suppressing zinc-finger protein A20 expression [96], may be relevant to the retinal neurodegeneration experienced in glaucoma patients from excited microglia [97]. Conversely, members of the miR-146 family are known negative regulators of the inflammatory pathway NF-κB through the suppression of thrombin-induced GPCR-mediated NF-κB activation in human retinal endothelial cells and inhibition of diabetes-induced NF-κB activation and retinal microvascular in diabetic rat models [98,99]. Furthermore, reports show that miR-146 is involved in the suppressive roles of COX-2 and IL-1β expression in human endothelial cells, epithelial cells, fibroblasts, and monocytes [100,101,102]. IL-1β expression has been shown in human optic nerves and reported significantly elevated in AH samples of POAG patients compared to controls [103]. With significantly increased oxidative stress reported in the aqueous humor of POAG patients, downregulation of tear miR-146b may signal its dysregulation to be a component of POAG pathophysiology [104].

On the contrary, miR-16 has been associated with the inhibition of cell proliferation and the induction of apoptosis due to its involvement with the suppression of the anti-apoptotic Bcl-2 gene [80]. With an increased presence of cell apoptosis in the trabecular meshwork of POAG patients [105], the down-regulation of miR-16 may indicate an upstream regulatory attempt at preventing cell apoptosis during glaucoma [28]. While reduced expression of miR-126 was reported in the tears of POAG patients [28], elevated miR-126 expression was reported in murine models of glaucoma [106]. The study also found that miR-126 facilitates apoptosis of retinal ganglion cells by promoting the VEGF-Notch pathway in murine models of glaucoma [106]. However, another study reported that pretreating monkey chorioretinal vessel endothelial cells with miR-126-mimics sharply decreased VEGF expression levels [107]. 

## 7. Diabetic Macular Edema

Diabetic macular edema (DME) leads to the breakdown of the blood-retinal barrier, resulting in macular thickening and intra-retinal leakage of fluids, proteins, and lipids [108]. In a recent study, the tear expression of twelve tear miRNAs was significantly correlated with either a good or a poor response to anti-VEGF treatment in DME patients. The expression of miR-214-3p, miR-320d, and miR-874-3p was positively correlated with a decrease in retinal central subfield thickness (CST) in response to anti-VEGF treatment while miR-98-5p, miR-196b-5p, and miR-454-3p correlated with increases in retinal CST following treatment [12]. Although limited by small group sizes (n = 6, n = 7, and n = 11), this study detected a greater number of unique miRNAs (331) in tears compared to serum (327) and aqueous humor samples (231).

miR-214 and miR-874 have been shown to suppress oxidative stress in diabetes and alleviate diabetes-induced nerve injury and inflammation [109,110,111]. The miR-320 family has been found to negatively regulate VEGF expression during high glucose exposure in human vein endothelial cells [112]. The upregulation of miR-320 in tear samples of DME patients may be in response to elevated glucose levels as studies have shown a correlation between the glucose content of plasma and tear fluid [113].

## 8. Diabetic Retinopathy

Diabetic retinopathy (DR) is a microvascular disease that can lead to irreversible vision impairment. As its prevalence continues to grow, there is a clear need for improved early detection and diagnosis. Tear miRNA composition has been shown to be altered in DR patients. Sun et al. found that miR-23a expression was decreased in the tears and serum of patients with type 2 diabetes or pre-diabetes compared to controls [18]. This is intriguing as miR-23a has been linked to a potential inhibitory role on VEGF, an important factor in the development of DR and blood vessel growth [114,115]. Hu et al. found elevated expression of miR-9-5p, miR-143-3p, miR-411-5p, miR-218-5p, miR-139-5p, miR-10b-5p, miR-129-5p, miR-214-3p, miR-381-3p, miR-130b-4p, miR-382-5p, miR-145-5p, miR-379-5p, miR-127-3p, miR-196a-5p, miR-206, miR-1-3p, miR-199a-5p, miR-642a-3p, and miR-642b-5p and decreased expression of miR-361-3p, miR-29c-5p, miR-532-5p, miR-3615, miR-429, miR-224-5p, miR-146a-5p, miR-149-5p, miR-339-3p, miR-203a-3p, miR-24-1-5p, miR-203b-5p, miR-31-5p, miR-147b-3p, miR-205-5p, miR-186-5p, miR-7706, miR-191-5p, miR-135b-5p, and miR-96-5p in tears of DR patients compared to healthy controls. It was also reported that the expression of miR-218-5p continuously increased from healthy controls to diabetic patients with and without DR and may be a potential biomarker for disease progression [27]. Sun et al. pre-selected miRNA-23a for RT-PCR analysis, while Hu et al. used RNA-Seq. However, miR-23a was not found to be decreased in the RNA-Seq study. Several factors may have led to this discrepancy. It is also important to mention that, while the mean ages of DR patients were similar between studies (53.6 vs. 54.6 years old), the mean age of the healthy controls differs (30.3 vs. 55.2 years old), which could impact the findings. Additionally, proteomic studies have found that the different tear sample collection techniques used in these two studies (Schirmer strips vs. capillary tubes) impact the identified tear composition [116]. It is possible that different collection methods have similar effects on identified miRNA content.

miR-145-5p expression has been shown to be elevated in high glucose-induced retinal ganglion cells (RGC) and linked to increased pro-inflammatory cytokines, IL-6, and apoptosis [117]. Another study found silencing of miR-145-5p inhibited apoptosis of RGCs via TRIM2-mediated activation of the PI3K/AKT signaling pathway in glaucoma rat models [118]. However, other literature has reported decreased expression of miR-145 in high glucose-treated retinal endothelial cells, making it apparent that more studies are needed to uncover miR-145’s role in various cell types [119]. Both miR-218-5p and miR-9-5p have been linked to insulin pathways [120,121]. miR-9 has also been reported to be significantly elevated in the serum of diabetic patients compared to controls and may serve as a potential biomarker for diabetes susceptibility [122]. Interestingly, miR-146 expression was also reportedly downregulated in the tears of glaucoma patients, but upregulated in the tears of HEK patients. The involvement of miR-146 in inflammatory pathways may signal dysfunctional inflammatory mechanisms that propagate DR or POAG symptoms as opposed to the herpes-induced complications found in HEK. Intraocular injection of pre-miR-31 has been shown to reduce retinal neovascularization and its absence in tears of DR patients may signal dysregulated pathways leading to its development [123]. Elevated miR-96 expression in RGC-5 cells showed significant hindrance to RGC-5 survival rates, and its reported decrease in DR tears may correlate with an attempt to prevent further damage [124].

## 9. Alzheimer’s Disease

Alzheimer’s disease (AD) is a neurodegenerative disease characterized by the progressive impairment of cognitive function [125]. Alterations in tear composition and flow rate have been associated with AD, and certain miRNAs, such as miR-15, have been linked to the inhibition of tau phosphorylation in mouse neuronal cells [16,126,127]. A recent study compared the tear miRNA profile of AD patients to healthy controls and found overlap of 133 miRNAs common between sample groups, of which 90 displayed altered expression levels. One miRNA, miR-200b-5p, was selected as a potential biomarker as it was exclusively detected in tear samples from AD patients [16]. Interestingly, miR-200b/c has been shown to be upregulated with the reduction of amyloid-β secretion and amyloid-β–induced memory impairment in primary neurons of AD murine models [128]. Another study showed the downregulation of miR-200b in the hippocampi of amyloid-precursor protein transgenic mice compared to controls [129]. The detection of miR-200b-5p in the tears of AD patients provides a potential biomarker and therapeutic target. 

## 10. Breast Cancer

While there have been great improvements in the diagnosis and treatment of metastatic breast cancer over the years, 5-year survival remains low [130,131]. Detection at the earliest stages is paramount to preventing disease progression, and the search for novel diagnostic methods continues to expand. miRNAs have become widely accepted as important components in a variety of pathways involved in regulating breast cancer [132]. However, non-invasive means of collection and quantification are limited. A study reported the expression of both miR-21 and miR-200c to be significantly higher in tear exosomes of patients with metastatic breast cancer compared to healthy controls [32]. It should be noted that the authors pre-selected these miRNAs for tear analysis as upregulation of these miRNAs has also been observed in the plasma of metastatic breast cancer patients compared to subjects with localized breast cancer and healthy controls [133,134,135]. A comprehensive tear miRNA profiling of metastatic breast cancer patients may provide additional miRNA biomarkers. 

miR-21 is frequently overexpressed in various human tumors [136], and functional studies have reported its involvement in oncogenic processes such as high proliferation [137], low apoptosis [138], and metastatic potential [139]. Interestingly, another study reported a strong association of elevated serum miR-200c expression with colorectal cancer metastasis [140]. Jurmeister et al. showed elevated miR-200c levels repress the migration and invasion of human breast cancer cells by targeting actin-regulatory proteins [141]. This may correlate to the elevated detection of miR-200c in the tears of metastasis breast cancer patients. 

## 11. Overall Summary

Compared to other biofluids like blood, serum, cerebrospinal fluid, saliva, and synovial fluid, a limited number of studies have been conducted so far on tear film miRNAs. With recent advances in detection methods and bioinformatics tools, studies exploring tear film miRNAs are gaining more traction due to their extracellular stability, tissue specificity, and posttranslational regulatory capabilities. As easily accessible biomarkers, tear miRNAs can provide a necessary window into the homeostatic state of the body and offer both biomarker and therapeutic potential. The present review provided an insight into the current findings of tear film miRNAs and their association with dry eye disease, Sjögren’s syndrome, herpes epithelial keratitis, vernal keratoconjunctivitis, primary open-angle glaucoma, diabetic macular edema, diabetic retinopathy, Alzheimer’s, and metastatic breast cancer. A complete list of the tear miRNAs and their biomarker potential in relation to these diseases is shown in Table 2.

## 12. Concluding Remarks and Future Directions

Due to the limited amount of research in the field, it is difficult to establish the baseline miRNA profile of human tears. The lack of standardized methods limit reproducibility and hinder the comparison of results between studies. Consequently, further studies using both discovery and targeted workflows are needed to uncover the biomarker potential of tear miRNAs.

Further, the expression and function of these molecules are known to be tissue-specific, which makes it challenging to extrapolate their regulatory mechanisms across a variety of tissues. Interpersonal differences and daily fluctuations of miRNAs also pose issues for controlled studies. The maturation of this field relies on the establishment of a proven means of collection, extraction, and analysis that can reduce the variability between studies and make large-scale collaboration feasible. Extensive examination of patient medical history can facilitate better categorization of samples, which will allow the identification of fluctuations in miRNA levels due to demographic variation and different stages of disease. Promoting follow-up studies with larger cohorts will help overcome the analytical difficulties posed by variability in miRNA expression. In these circumstances, effective sampling and analytical techniques are crucial, and improving data storage and sharing is essential to establishing the framework for tear miRNAs as biomarkers.

## Figures and Tables

**Table 1:** Summary of literature reporting tear microRNAs as potential biomarkers.

Study	Disease	Number of Participants	Tear Collection Method	Detection Method
Wang, Q. et al. (2020) [21]	Dry eye Disease (DED)	138 HC; 138 DED	Capillary tube	miRNA microarray
Pucker, A.D. et al. (2022) [29]	Dry Eye Disease (DED)	5 HC; 5 DED	Phosphate buffer saline (PBS) wash	RNA-Seq
Kim, Y.J. et al. (2019) [19]	Sjögren Syndrome (SS)	8 HC; 18 SS	Micropipettes	RT-PCR
Kim, Y.J. et al. (2022) [22]	Herpes Epithelial Keratitis (HEK)	7 HC; 8 HEK	Micropipettes	RT-PCR
Syed, N.H. et al. (2022) [31]	Vernal Keratoconjunctivitis (VKC)	4 HC; 4 VKC	Schirmer strip	miRNA microarray
Tamkovich, S. et al. (2019) [28]	Primary Open Angle Glaucoma (POAG)	29 HC; 33 POAG	Capillary tube	RT-PCR
Raga-Cervera, J. et al. (2021) [20]	Primary Open Angle Glaucoma (POAG)	22 OHT; 20 POAG	Capillary tube	RNA-Seq
Chan, H.W. et al. (2020) [12]	Diabetic Macular Edema (DME)	24 DME	Schirmer strip	RT-PCR
Sun, L. et al. (2021) [18]	Diabetic Retinopathy (DR)	28 HC; 33 DR	Capillary tube	RT-PCR
Hu, L. et al. (2022) [27]	Diabetic Retinopathy (DR)	11 HC; 10 DM; 9 DR	Schirmer strip	RNA-Seq
Kenny, A. et al. (2019) [16]	Alzheimer’s Disease (AD)	15 HC; 8 MCI; 9 AD	Schirmer strip	RT-PCR
Inubushi, S. et al. (2020) [32]	Metastatic Breast Cancer (MBC)	8 HC; 5 MBC	Schirmer strip	RT-PCR

HC = Healthy control; OHT = Ocular hypertension; DM = Diabetes mellitus; MCI = mild cognitive impairment.

**Table 2:** Up and down-regulated tear microRNAs reported as potential biomarkers.

Condition	Tear miRNAs in Disease State vs. Controls	
	Upregulated	Downregulated
Dry Eye Disease	miR-127-5p, miR-1273h-3p, miR-1288-5p, miR-130b-5p, miR-139-3p, miR-1910-5p, miR-203b-5p, miR-22-5p, miR-4632-3p, miR-450b-5p, miR-1283, miR-5700, miR-3671	miR-4673, miR-890, miR-576-5p, miR-337-3p, miR-607, miR-1827, miR-485-5p, miR-6884-5p, miR-335-3p, miR-647, miR-4433b-5p, miR-570-3p, miR-1276, miR-2054, miR-802, miR-539-5p, miR-198, miR-3646, miR-4427, miR-4705, miR-606, miR-20b-3p, miR-4475, miR-4478, miR-4797-5p, miR-142-5p, miR-153-5p, miR-5583-5p
Sjögren’s Syndrome	miR-16-5p, miR-34a-5p, miR-142-3p, miR-223-3p	miR-30b-5p, miR-30c-5p, miR-30d-5p, miR-92a-3p, miR-134-5p, miR-137, miR-302d-5p, miR-365b-3p, miR-374c-5p, miR-487b-3p
Herpes Epithelial Keratitis	miR-15b-5p, miR-16-5p, miR-20b-5p, miR-21-5p, miR-23b-3p, miR-25-3p, miR-29a-3p, miR-30a-3p, miR-30d-5p, miR-92a-3p, miR-124-3p, miR-127-3p, miR-132-3p, miR-142-3p, miR-145-5p, miR-146a-5p, miR-146b-5p, miR-155-5p, miR-182-5p, miR-183-5p, miR-221-3p, miR-223-3p, miR-338-5p	
Vernal Keratoconjunctivitis	miR-1229-5p, miR-6821-5p, miR-6800-5p, miR-4466, miR-3665, miR-4530, miR-7110-5p, miR-1207-5p, miR-6875-5p, miR-762, miR-4741, miR-6740-5p, miR-4298, miR-7107-5p, miR-2861, miR-3663-3p, miR-6891-5p, miR-4672, miR-6785-5p, miR-6510-5p, miR-6803-5p, miR-718, miR-642b-3p, miR-6124, miR-4687-3p, miR-4721, miR-4459, miR-8072, miR-5703, miR-3195, miR-7847-3p, miR-4665-3p, miR-6869-5p, miR-638, miR-6087, miR-4516, miR-3960, miR-6089, miR-4443, miR-5787, miR-642a-3p, miR-3679-5p, miR-6088, miR-8069, miR-4281, miR-1273g-3p, miR-7150, miR-940	miR-7975, miR-7977, miR-1260a
Primary Open-Angle Glaucoma	miR-26b-5p, miR-27a-3p, miR-152-3p, miR-30e-5p, miR-125b-2-5p, miR-224-5p	miR-151a-3p, miR-1307-3p, miR-146b, miR-16, miR-126
Response to Anti-VEGF Treatments for Diabetic Macular Edema	Correlated with good response: miR-214-3p, miR-320d, miR-874-3p, Correlated with poor response: miR-98-5p, miR-196b-5p, miR-454-3p	
Diabetic Retinopathy	miR-9-5p, miR-143-3p, miR-411-5p, miR-218-5p, miR-139-5p, miR-10b-5p, miR-129-5p, miR-214-3p, miR-381-3p, miR-130b-4p, miR-382-5p, miR-145-5p, miR-379-5p, miR-127-3p, miR-196a-5p, miR-206, miR-1-3p, miR-199a-5p, miR-642a-3p, miR-642b-5p	miR-23a, miR-361-3p, miR-29c-5p, miR-532-5p, miR-3615, miR-429, miR-224-5p, miR-146a-5p, miR-149-5p, miR-339-3p, miR-203a-3p, miR-24-1-5p, miR-203b-5p, miR-31-5p, miR-147b-3p, miR-205-5p, miR-186-5p, miR-7706, miR-191-5p, miR-135b-5p, miR-96-5p
Alzheimer’s Disease	miR-200b-5p	
Metastatic Breast Cancer	miR-21, miR-200c	

## Data Availability

Data sharing not applicable.
